# Supplementary material for: A Humanized Yeast Phenomic Model of Deoxycytidine Kinase to Predict Genetic Buffering of Nucleoside Analog Cytotoxicity
Source: Genes (Basel). 2019 Sep 30;10(10):770. doi: 10.3390/genes10100770 (PMC6826991; doi:10.3390/genes10100770)

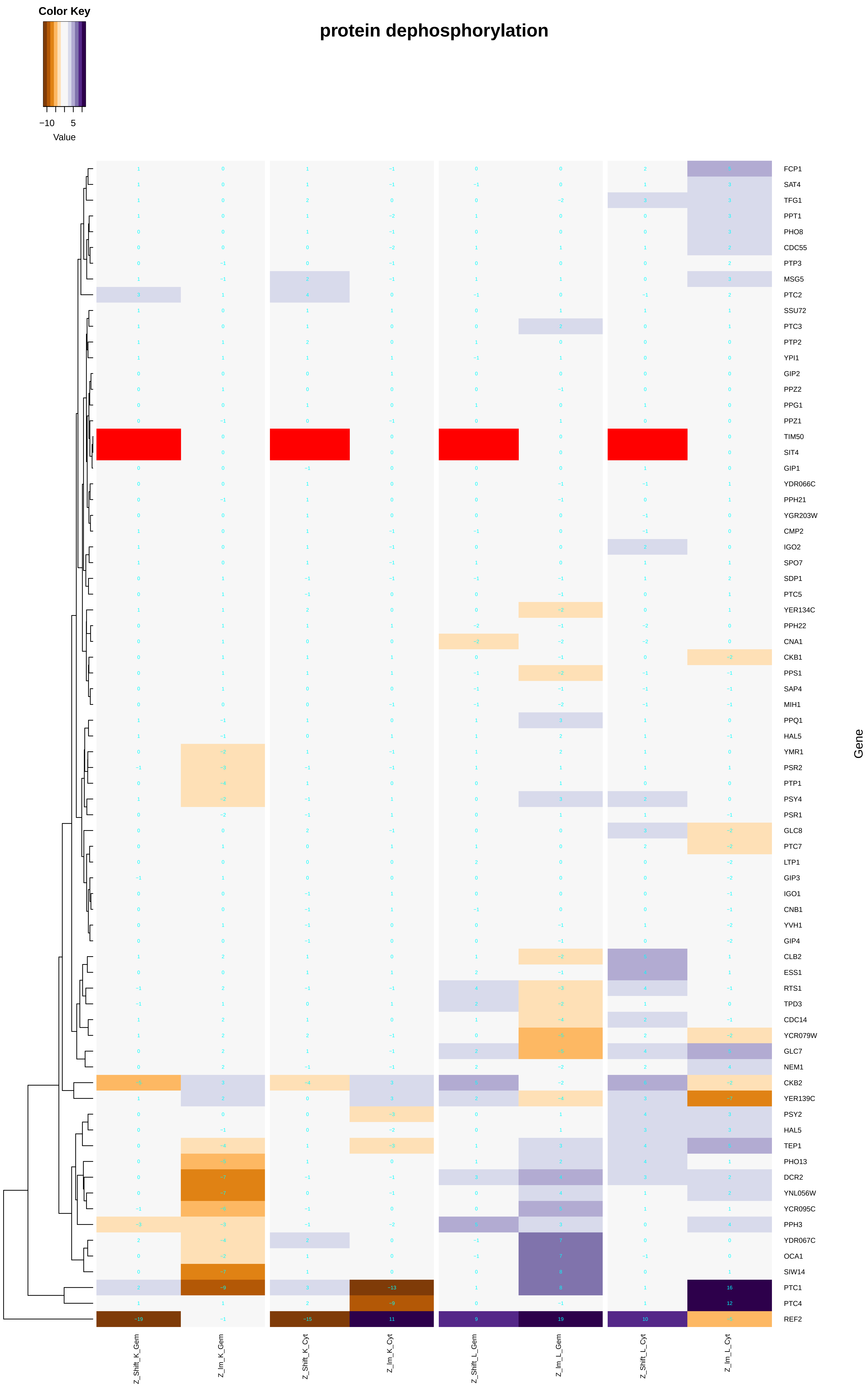

protein dephosphorylation
Color Key
−10
5
Value
FCP1
SAT4
TFG1
PPT1
PHO8
CDC55
PTP3
MSG5
PTC2
SSU72
PTC3
PTP2
YPI1
GIP2
PPZ2
PPG1
PPZ1
TIM50
SIT4
GIP1
YDR066C
PPH21
YGR203W
CMP2
IGO2
SPO7
SDP1
PTC5
YER134C
PPH22
CNA1
CKB1
PPS1
SAP4
MIH1
PPQ1
HAL5
YMR1
PSR2
PTP1
PSY4
PSR1
GLC8
PTC7
LTP1
GIP3
IGO1
CNB1
YVH1
GIP4
CLB2
ESS1
RTS1
TPD3
CDC14
YCR079W
GLC7
NEM1
CKB2
YER139C
PSY2
HAL5
TEP1
PHO13
DCR2
YNL056W
YCR095C
PPH3
YDR067C
OCA1
SIW14
PTC1
PTC4
REF2
Gene
Z_Shift_K_Gem
Z_Im_K_Gem
Z_Shift_K_Cyt
Z_Im_K_Cyt
Z_Shift_L_Gem
Z_Im_L_Gem
Z_Shift_L_Cyt
Z_Im_L_Cyt

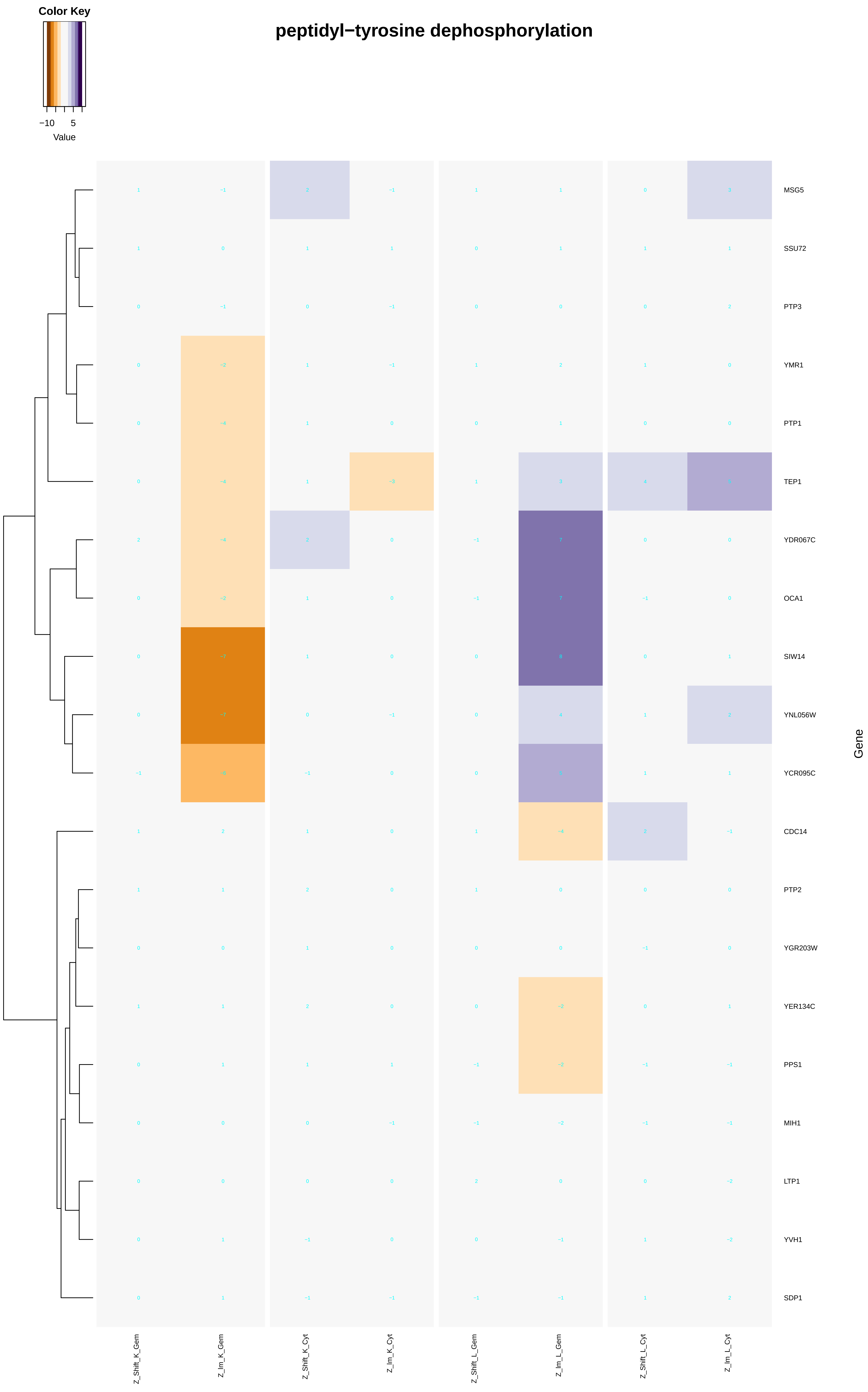

peptidyl−tyrosine dephosphorylation
Color Key
−10
5
Value
MSG5
SSU72
PTP3
YMR1
PTP1
TEP1
YDR067C
OCA1
SIW14
YNL056W
YCR095C
CDC14
PTP2
YGR203W
YER134C
PPS1
MIH1
LTP1
YVH1
SDP1
Gene
Z_Shift_K_Gem
Z_lm_K_Gem
Z_Shift_K_Cyt
Z_lm_K_Cyt
Z_Shift_L_Gem
Z_lm_L_Gem
Z_Shift_L_Cyt
Z_lm_L_Cyt

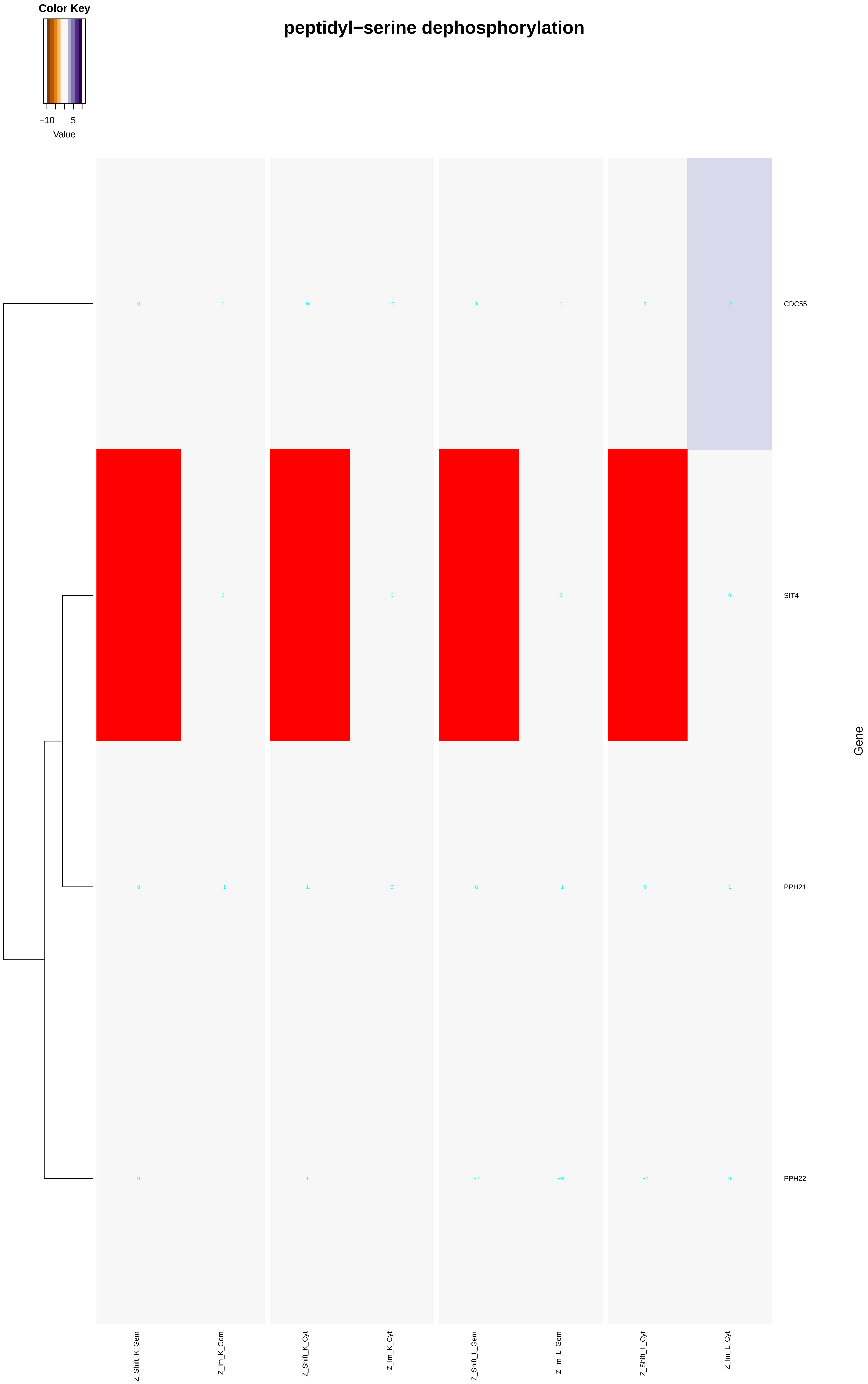
Color Key
−10
5
Value
peptidyl−serine dephosphorylation
0
0
0
−2
1
1
1
2
CDC55
0
0
0
0
SIT4
0
−1
1
0
0
−1
0
1
PPH21
0
1
1
1
−2
−1
−2
0
PPH22
Gene
Z_Shift_K_Gem
Z_Im_K_Gem
Z_Shift_K_Cyt
Z_Im_K_Cyt
Z_Shift_L_Gem
Z_Im_L_Gem
Z_Shift_L_Cyt
Z_Im_L_Cyt

dephosphorylation of RNA polymerase II C−terminal domain

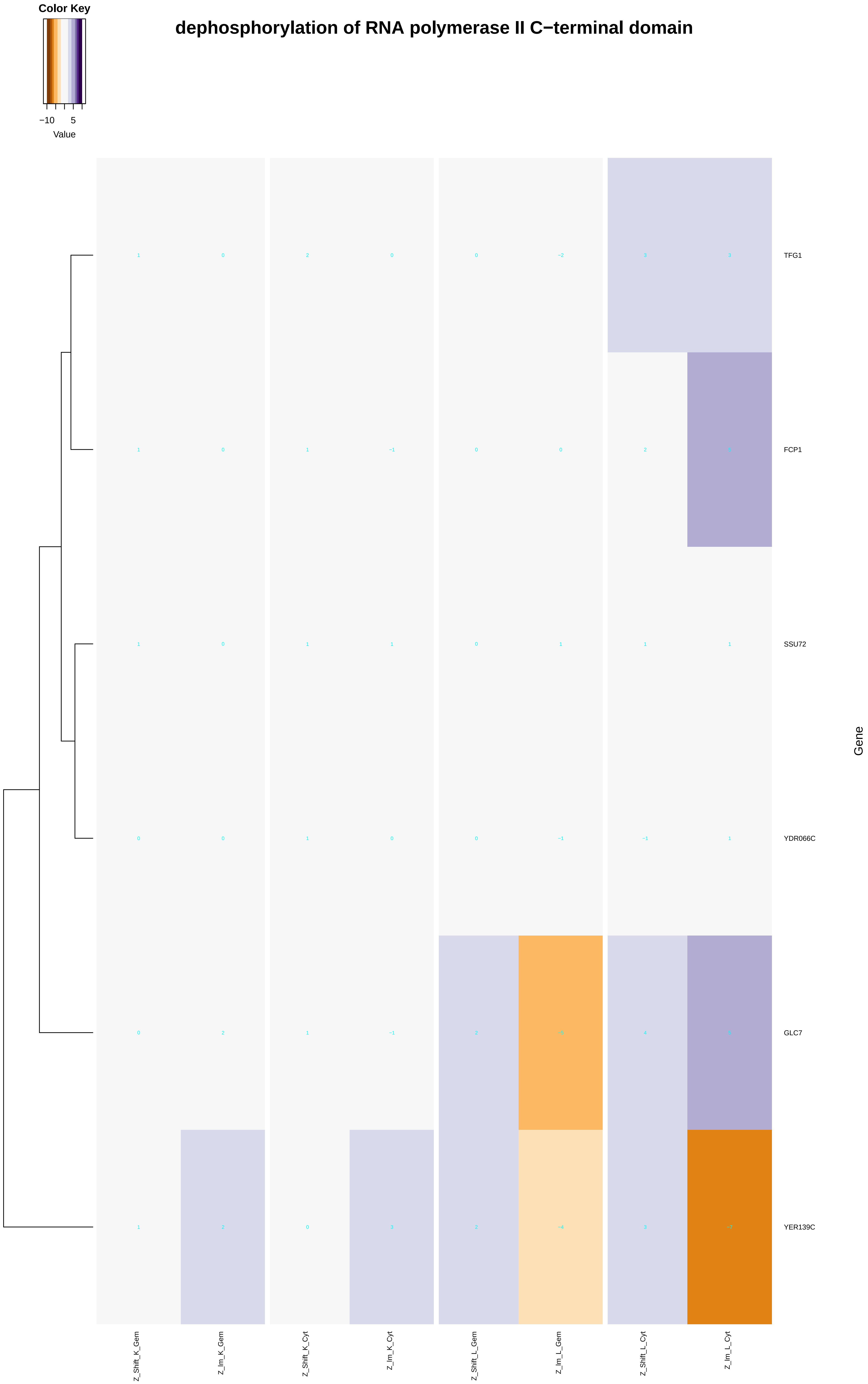

Supplement: Supplementary file 1 [file genes-10-00770-s001.zip › Additional_Files/Additional_File7_GOTermHeatmaps.zip!/Additional_File7_GOTermHeatmaps/protein_dephosphorylation.pdf]
